# Gut-Microbiota, and Multiple Sclerosis: Background, Evidence, and Perspectives

**DOI:** 10.3390/nu15040942

**Published:** 2023-02-14

**Authors:** Clelia Altieri, Barbara Speranza, Maria Rosaria Corbo, Milena Sinigaglia, Antonio Bevilacqua

**Affiliations:** Department of Agriculture, Food, Natural Resources and Engineering (DAFNE), University of Foggia, 71122 Foggia, Italy

**Keywords:** gut microbiota, diet, multiple sclerosis, dysbiosis, probiotics

## Abstract

Many scientific studies reveal a significant connection between human intestinal microbiota, eating habits, and the development of chronic-degenerative diseases; therefore, alterations in the composition and function of the microbiota may be accompanied by different chronic inflammatory mechanisms. Multiple sclerosis (MS) is an inflammatory demyelinating disease of the central nervous system (CNS), in which autoreactive immune cells attack the myelin sheaths of the neurons. The purpose of this paper was to describe the main changes that occur in the gut microbiota of MS patients, with a focus on both microbiota and its implications for health and disease, as well as the variables that influence it. Another point stressed by this paper is the role of microbiota as a triggering factor to modulate the responses of the innate and adaptive immune systems, both in the intestine and in the brain. In addition, a comprehensive overview of the taxa modified by the disease is presented, with some points on microbiota modulation as a therapeutic approach for MS. Finally, the significance of gastro-intestinal pains (indirectly related to dysbiosis) was assessed using a case study (questionnaire for MS patients), as was the willingness of MS patients to modulate gut microbiota with probiotics.

## 1. Introduction

The human gastrointestinal tract contains an abundant microbial community, represented by more than 100 trillion microorganisms, making the colon one of the most densely populated microbial habitats known on earth. In fact, the gut microbiome encodes over 3 million genes, producing thousands of metabolites, whereas the human genome consists of approximately 23,000 genes [1]. The gut microbiome, because of its significance, definitely plays an important role in the health and disease of the host [2].

Many studies reveal a significant connection between human intestinal microbiota, eating habits, and the development of chronic-degenerative diseases, or more precisely, low-grade inflammation, which is the primary target to address in order to ameliorate the course of many diseases, as well as Multiple Sclerosis (MS).

Many functional and dysfunctional connections have been explained by virtue of the extent of the symbiosis between the human organism and the microbial population residing in various parts of the body, especially in the gastrointestinal tract. It is clear that the influence of the microbiota is not limited to local effects but also extends to remote organs, particularly the brain [3].

MS is an inflammatory demyelinating disease of the central nervous system (CNS), in which autoreactive immune cells attack the myelin sheaths of the neurons. Pro-inflammatory immune cells, such as T helper type 1 (Th1) and T helper type 17 (Th17) T-cells, primarily mediate the pathogenesis of MS by producing pro-inflammatory cytokines [4].

A growing spread of MS among young adults and the autoimmune nature of this disease, which affects the central nervous system (CNS), have been recently observed, and more details in the context of the two-way communication between the intestinal microbiota and the CNS (the gut-brain axis) are available.

Thus, the goal of this paper was to describe the main changes occurring in the gut microbiota of MS patients. Some reviews are available in the literature, but they offer a medical perspective with a focus on the etiology of this disease, the correlation of microbiota with some variables, and the importance of epigenetics.

Therefore, a new point of view was stressed, namely the point of view of microbiologists, focusing on gut microbiota and its implications on health and disease, as well as the variables affecting it. Another point stressed by this paper is the role of microbiota as a triggering factor to modulate the responses of the innate and adaptive immune systems, both in the intestine and in the brain. The focus was on microbial metabolites derived from the processing of fibers and the essential amino acid tryptophan. Then, a focus on the gut-brain axis was offered to highlight the importance of this topic in connection to MS.

In addition, the importance of gut microbiota for MS was stressed, offering for the first time a comprehensive overview of the taxa modified by the disease as well as some points on microbiota modulation as a therapeutic approach for MS. Finally, the importance of gastro-intestinal pains (indirectly related to dysbiosis) was assessed using a questionnaire for MS patients, as was their willingness to modulate gut microbiota with probiotics.

From a methodological point of view, the effects of MS on gut microbiota, dysbiosis and therapeutic approaches based on microbiota were studied through papers selected on Scopus, WoS (Web of Science), and PubMed, setting a temporal range of ca. 10 years, although older papers were cited if they could offer an overview on basic mechanisms. A wide variety of keywords was used, including gut microbiota and multiple sclerosis, dysbiosis in multiple sclerosis, modulation of gut microbiota, microbiota and autoimmune diseases, gut-brain axis, microbial metabolites, triggering factors, and diet. Papers offering only hypotheses or results based only on animal models, without a clear connection with human gut microbiota, were excluded.

## 2. Gut Microbiota

Gut microbiota is a complex ecosystem, and each individual harbors a different microbiota; however, there are dominant phyla mainly represented by Bacteroidetes (~20–25%), Firmicutes (~60–65%), Proteobacteria (~5–10%), and Actinobacteria (~3%), which together constitute over 97% of the gut microbe population. The taxonomic variability in the human gut is higher than the functional variability, as measured by a variety of methods, suggesting that many different configurations of the microbiota lead to essentially the same functional result [5].

The Firmicutes phylum includes more than 200 genera, but the most representative are *Clostridium*, *Lactobacillus*, *Bacillus*, *Enterococcus*, and *Ruminicoccus*. In the phylum Bacteroidetes, the preponderant genera are *Bacteroides* and *Prevotella*. The phylum Actinobacteria is mainly represented by the genus *Bifidobacterium* [1]. Supplementary Table S1 gives some details on the composition of gut microbiota, as well as on the role of the different phyla in the host’s physiology.

Gut microbiota can be classified in terms of enterotypes as follows:

1. Enterotype 1: the dominant genus is *Bacteroides*, followed by *Parabacteroides*. These two genera show abundant expression of some enzymes, like galactosidase, hexosaminidase, and protease. Individuals with enterotype-1 can recover the most energy from the fermentation of carbohydrates and proteins; as a result, they are accustomed to a Western-style diet. This enterotype shows a higher production of riboflavin, pantothenic acid, and biotin (B2–B5–B8) compared to the others. Such a composition of the gut microbiota often correlates with a general inflammatory condition.
2. Enterotype 2: the dominant genera are *Prevotella* and *Desulfovibrio*; they can produce high levels of thiamine (B1) and folic acid (B9). Therefore, this enterotype is the result of a purely vegetarian diet.
3. Enterotype 3: the dominant genera are *Ruminococcus* and *Akkermansia*, which prefer simple sugars but can degrade mucin. These microorganisms can unbalance the normal functioning of the immune system [6].

**Figure 1:**
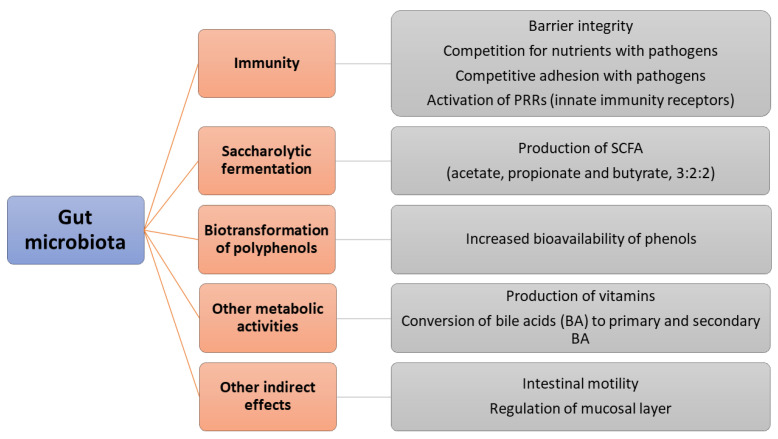
Some functions of gut microbiota [7,8,9,10,11,12,13,14]. SCFA are short-chain fatty acids.

Literature reports many roles for gut microbiota, dealing with different pathways. Figure 1 shows a synopsis of the most important functions worldwide attributed to it.

The composition of gut microbiota varies along the digestive tract in relation to pH, O_2_ concentration, digestive flow rate and secretions; moreover, it is strongly affected by internal and external factors, biotic and abiotic factors. Some of these factors could cause or contribute to an unbalanced composition and/or disequilibrium of gut microbiota, known as dysbiosis, and usually related to a variety of diseases.

The environmental input influencing gut microbiota may be represented by physical activity, diet, and circadian rhythm. They have been shown to drive changes in microbial profiles and activities, such as the production of fermentation end-products, SCFA, and the biotransformation of dietary compounds and xenobiotics [15,16,17].

Also, ethnicity makes the gut a very dynamic microbial system. Ethnicity could be considered a macro-variable, including different factors, e.g., place, eating habits, way of food preparation, habits in terms of additives and chemicals used, lifestyle, etc. [18,19]. For example, two studies showed that the African gut microbiota clearly has the enterotype *Prevotella* (enterotype 2), due to a large consumption of millet/sorghum and other local vegetables containing very few lipids and animal proteins, while the European gut microbiota (Western diet) shows mainly the enterotype *Bacteroides* (enterotype 1), influenced by a diet rich in lipids and animal proteins [20,21]. Syromyatnikov et al. [22] addressed this issue and highlighted some key features of the gut microbiota of people from Africa, America, Asia, and Europe. The microbiota of European individuals is generally composed of (in order of importance) Firmicutes, Bacteroidota, Actinobacteria, Proteobacteria, Fusobacteria, and Verrucomicrobia, while the analysis of gut microbiota from African people revealed the dominance of *Prevotella*, with a possible effect of sex in a study on individuals from Tanzania (prevalence of *Treponema* in women and *Eubacterium*/*Blautia* in men) [21]. A study conducted on immigrants in San Francisco Bay revealed an imprinting in the microbiota of Caucasians (*Akkermansia muciniphila*, *Bacteroidales bacterium* ph8, and *Roseburia hominis*), while the microbiome of East-Asian members had a higher abundance of *Ruminococcus gnavus* [23]. Finally, the microbiota of Japanese people was completely different, with at least 5 different models labeled as types A (abundance of *Prevotella*), B (*Bacteroides*, *Blautia*, and *Faecalibacterium*), C (*Bacteroides*, *Fusobacterium*, and *Proteus*), D (*Bifidobacterium*), and E (*Prevotella*) [24].

The role of ethnicity is not clear because there are many confounding factors or variables that could reduce or increase its weight, like lifestyle (rural vs. urban housing context) [20], religion [25,26,27,28,29,30,31], diseases [32,33], and physical activity (high-intensity exercises vs. endurance, sedentary vs. physical activity) [32]. The most stressful factor is probably diet, as it is well known that diet styles and nutrients directly affect the qualitative and quantitative composition of gut microbiota. For example, a Western diet determines a reduction in abundance of *Akkermansia muciniphila*, *Faecalibacterium prausnitzii*, *Roseburia* spp., *Eubacterium* spp., and *Clostridium* clusters XIVa and IV [34,35], while some authors report an increase in the ratio P/B (*Prevotella*/*Bacteroides*) in a vegan diet [36,37].

## 3. Gut-Brain Axis: Role of Microbiota

A gut-brain axis (GBA) represents a dense and complex system of bidirectional communication existing between the gut microbiota and the central nervous system (CNS). This system is divided into 3 components: the neuronal connections, the general neuroendocrine and humoral pathways, and the immune system. The CNS can communicate directly with the intestine via sympathetic or parasympathetic branches of the autonomic nervous system (ANS), particularly via the vagus nerve. Hence, the microbiome can be modulated directly by bioactive molecules released by the enteric nervous system (ENS) or indirectly through other mechanisms that modify the microbial environment, such as gastrointestinal motility, permeability, pH value, or mucus secretion [38].

These adjustments are mostly mediated by the ANS’s secretion of acetylcholine or catecholamines, mediators influencing the circuits of the enteric nervous system.

For example, some studies suggest that acetylcholine is able to suppress the secretion of pro-inflammatory factors such as tumor necrosis factor alpha (TNFα), interleukin 6 (IL-6), and IL-18. Experiments with vagotomized mice illustrated the critical role of the vagus nerve in the dialogue between the gastrointestinal tract and the CNS. Treatment of mice with *Lacticaseibacillus rhamnosus* reduced the expression of the γ-aminobutyric acid (GABA) receptor in the brain and thus induced anxiolytic and antidepressant effects, that disappeared in mice after vagotomy. Similarly, no anxiolytic and behavioral influence was detected by *Bifidobacterium longum* in vagotomized mice with chronic colitis, while an attenuation of psychological comorbidities of colitis was observed after administration of *Bifidobacterium longum* in mice with a nerve signal by intact vagus [39].

The microbial metabolites (propionic acid, butyric acid, and acetic acid) also act as indirect neuromodulators; for example, in the intestine, serotonin is released by enterochromaffin cells (ECC) after their stimulation by the SCFAs [40].

Serotonin, or 5-hydroxytryptamine (5-HT), is synthesized from the amino acid tryptophan (5-HTP). The intestinal microbiota regulates the peripheral availability of Trp and therefore the synthesis of serotonin also at the level of the CNS; in fact, some studies conducted on germ free mice show increased levels of plasma Trp and hippocampal 5-HT after intestinal bacterial colonization [41].

Although the exact mechanisms of peripheral regulation of Trp are unknown, some studies indicate that the microbiota modulates the degradation of Trp along the kynurenine pathway.

Tryptophan can also be metabolized by the intestinal microbiota into indole and its derivatives, such as indole-3-aldehyde (IAld), indole-3-acetic acid (IAA), and indole-3-propionic acid (PAH). Indole formation from tryptophan occurs through activation of the tryptophanase (TnaA) enzyme, which can be found in many gram-negative and gram-positive bacterial species, including *Escherichia coli*, *Clostridium* spp., and *Bacteroides* spp. [41].

Beneficial anti-inflammatory effects are attributed to IPA, IAId, and IAA. These three metabolites of tryptophan would carry out the anti-inflammatory action as ligands of the aryl receptor for hydrocarbons (AHR) [8]. In particular, if these metabolites bind AHR receptors expressed on astrocytes, there is a significant reduction in inflammation of the CNS [41].

The neuroendocrine communication between intestinal microbiota and brain involves the Hypothalamus-Pituitary-Adrenal axis (HPA); in fact, glucocorticoids, mineralocorticoids or catecholamines, released following the activation of the HPA axis, can alter the composition of the microbiota, the permeability of the intestinal epithelium and the immune responses of the intestinal mucosa.

Many scientific studies have demonstrated that, from birth, the development of the immune system depends significantly on the biodiversity of the intestinal microbiota [42].

Contact with microbes is therefore essential for the development of efficient immunity, both at peripheral level and at intestinal level, for the production of GALT (lymphoid tissue associated with the intestine).

Gut microbial metabolites play a key role in inflammatory signaling, interacting both directly and indirectly with the host’s immune cells. SCFAs perform many of their actions by interacting with certain G-protein-coupled receptors (GPRs).

Acetate, a SCFA highly produced by bifidobacteria, regulates intestinal inflammation by stimulating the GPR43 receptor. In particular, when acetate binds to this receptor, it seems to inhibit the secretion of pro-inflammatory IL-18, of which high levels are found in the case of colon cancer [43].

Maslowski et al. [44] found that activation of GPR43 by SCFAs was necessary for normal resolution of some inflammatory responses; in fact, mice deficient in GPR43 (Gpr43 −/−) were shown to be unable to resolve inflammation in models of colitis, arthritis and asthma [45].

## 4. Multiple Sclerosis and Gut Microbiota

According to Compston and Coles [46], "Multiple sclerosis (MS) is primarily an inflammatory disorder of the brain and spinal cord in which focal lymphocytic infiltration leads to damage of myelin and axons." Autoantibodies cause the formation of circumscribed areas of demyelination in the brain and spinal cord, also called plaques. The pathological features of the plaques are the rupture of the blood-brain barrier (BBB), damage to the myelin sheath, oligodendrocytes, and axons, and an inflammatory infiltrate consisting mainly of lymphocytes and macrophages.

MS has a multifactorial pathogenesis, for which the dysfunctionality of the immune system (IS) has multiple causes, such as genetic predisposition, exposure to viral infectious agents, possible heavy metal poisoning, and pro-inflammatory lifestyles [47,48].

In about 85% of patients, MS occurs in a relapsing-remitting form (RRMS), 4% of people are diagnosed with secondary progressive multiple sclerosis (SPMS), and 10% are diagnosed with primary progressive multiple sclerosis (PPMS). If the RR-MS form is not treated in 50–60% of cases, it evolves towards the primary-progressive form.

Finally, a small percentage of people manifest a form of progressive multiple sclerosis immediately, with occasional relapses (relapsing—progressive multiple sclerosis).

The RRMS type is characterized by periods when symptoms are evident (relapse), followed by periods when symptoms disappear or improve (remission). In the case of SPMS and PPMS, there are no obvious relapses, but there is a gradual worsening of the disability over time.

The risk of developing MS is driven by both genetic factors and environmental exposures. The main genetic risk loci are in the HLA region (risk allele HLA-DRB1*15:01) [49]. However, more than 200 different DNA variants associated with disease susceptibility have been identified [50].

In addition, epigenetics (heritable or non-heritable changes not related to modified DNA sequences and leading to an altered expression or translation of the genome) also plays a major role in MS; the most important mechanisms are DNA methylation, histone modification, and RNA interference [51]. The goal of this paper was to discuss microbiota, and the medical traits of MS are outside the expertise of the authors, who are microbiologists. However, some notes could be useful for a better understanding of the pathology’s onset.

The importance of epigenetics in MS has been elucidated through studies on twins, and the data available suggest that the mechanisms are similar to those found in other autoimmune diseases (DNA methylation, histone modifications, and miRNA-based gene expression regulation); they promote a proinflammatory response, while few data are available on how epigenetics acts on other MS-associated factors (mitochondrial dysfunction, oxidative stress, and axonal degeneration) [51]. Several studies reported either decreased (HERV-W and PAD2) or increased methylation (SHP-1), increased histone citrullination (PAD4), increased (miR-17 and miR-20a), or decreased expression (among others, miR-155) [51]. There is also evidence that epigenetic changes occur in experimental autoimmune encephalomyelitis (EAE) studies [51].

MS is also triggered by environmental risk factors, including smoking, reduced exposure to sunlight, and infection with the Epstein–Barr virus [52]. For example, tobacco smoking is generally associated with an increased risk of MS in a dose-dependent manner [52], probably due to the gene-environment interaction "smoking and HLA-related MS susceptibility" [53], depending on lung irritation due to smoke inhalation and the triggering of T cells in the lungs [54]. Moreover, smoking is probably related to a worse diagnosis and a faster progression of the disease [52]. Another important environmental factor is low sun exposure, which causes a deficiency in vitamin D [55], probably due to the role of this vitamin in the regulation of inflammatory phenomena in auto-immune diseases [56]. Also, obesity and a high BMI index in adolescence increase the risk of MS, probably linked to inactivity, which in turn is responsible for increased peripheral inflammation [57].

According to some studies, there is also an important association between atmospheric pollution and pediatric MS, attributed to prolonged exposure to particulate matter with a diameter of 2.5 μm (PM2.5), carbon monoxide (CO), lead, and sulfur dioxide (SO_2_) [58]. Januel et al. [59] showed a significant odds ratio between exposure to PM2.5 and recurrences of MS, particularly in patients under the age of 30. The same study describes the mechanism by which PM2.5 would induce the (re)activation of the disease, i.e., the fine particulate reaches the pulmonary alveoli, activating the macrophages and causing the release of pro-inflammatory cytokines that induce the differentiation of T lymphocytes into Th1 lymphocytes. Part of these pro-inflammatory Th1 lymphocytes migrate to the CNS, triggering the inflammatory process in situ and lending credence to the idea of a lung-brain axis. This lung-brain axis would also underline the relationship between MS and exposure to cigarette smoke [60].

## 5. Dysbiosis in Multiple Sclerosis Patients and Therapeutic Approaches

Dysbiosis may be associated with endotoxemia, intestinal/systemic inflammation, and trigger neuroinflammation, whereas a healthy gut microbiota may dampen the inflammatory processes with the production of antiinflammatory molecules [61,62,63,64].

Recently, intestinal microbiota emerged as an additional potential influencing or triggering factor for MS [65,66], as well as for other autoimmune diseases [67].

There is evidence in the literature that gut bifidobacteria play a role in the onset of MS. A protective role of bifidobacteria in the pathogenesis of MS—and also of Guillain-Barré syndrome (GBS)—was hypothesized by Shi et al. [68]: a lower level of bifidobacteria has been negatively correlated with the levels of IL-17A in cerebrospinal fluid (CSF) and plasma samples of patients. IFN-γ and IL-17 are produced by pro-inflammatory Th1 and Th17 cells, respectively, and have important roles in the pathogenesis of both MS and GBS [69]. Notably, GBS, like MS, is an autoimmune demyelinating disorder, and the coexistence of these two syndromes in a patient has been reported [70].

Studies using experimental models have indicated that multiple sclerosis (MS)-like disease can be triggered in the gut following interactions of brain autoimmune T lymphocytes with local microbiota. When the human-derived microbiota was transferred into transgenic mice expressing a myelin autoantigen-specific T cell receptor, it was found that gut microbiota from MS-affected twins induced central nervous system (CNS)-specific autoimmunity at a higher incidence than microbiota from healthy co-twins. So there was functional evidence that human microbiome components contribute to CNS-specific autoimmunity [71].

Specifically, differences in microbial abundance between MS patients and controls were observed, and it was investigated how particular MS-associated bacteria modulate T lymphocyte responses using both in vitro and in vivo model systems. There is evidence that differences in specific gut bacteria are functionally associated with a shift toward a pro-inflammatory T cell profile, exacerbating autoimmune responses and thus potentially contributing to MS pathogenesis [72].

Berer and collaborators [71] compared the composition of the gut microbiota in 34 pairs of monozygotic twins, which were discordant for MS. There were no differences in the quality and quantity of intestinal microorganisms, except for a higher presence of *Akkermansia* in the twins with MS not undergoing therapy and a higher relative abundance of *Sutturella* in healthy twins. In murine transplants, the microbiota of MS patients caused a significantly higher incidence of autoimmunity.

The results showed *Akkermansia* to be more abundant in mice transplanted with fecal material from the MS twins and *Sutterella* to be more abundant in mice that received fecal material from healthy twins.

It has been shown that in patients with MS, there is a lower presence of the genera *Bacterioides*, *Parabacteroides*, *Prevotella*, and *Lactobacillus*; on the other hand, there is a higher abundance of *Akkermansia*, *Ruminococcus*, *Blautia*, and *Bifidobacterium* (enterotype 3).

Cekanaviciute et al. [72] identified specific bacteria associated with MS and demonstrated that these bacteria regulate T-cell-mediated adaptive immune responses and create a pro-inflammatory environment in vitro and in vivo. The findings of these researchers may suggest “therapeutic” targeting of the microbiota as a treatment for MS. The microbiome of 71 individuals with RR-MS and 71 healthy controls highlighted in the microbiota of subjects affected by MS a greater presence of *Akkermansia muciniphila* and *Acinetobacter calcoaceticus* and a lower load of *Parabacteroides distasonis*.

**Figure 2:**
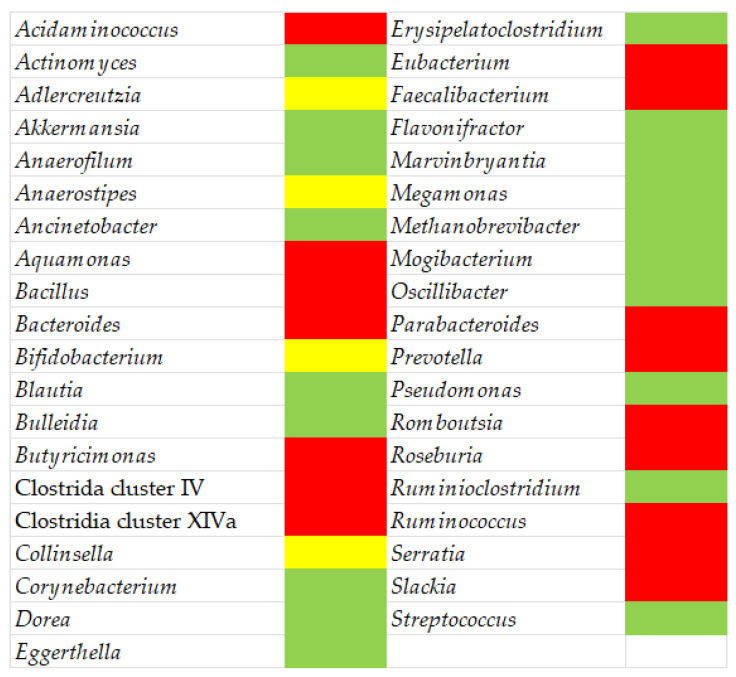
Gut microbiota in MS patients: **green**, higher abundance compared to healthy subjects; **red**, decreased abundance; **yellow**, conflicting results. Built by authors using data from various references.

Figure 2 depicts a synopsis of the gut microbiota and the differences between MS patients and healthy individuals.

Data and evidence available suggest that an action on gut microbiota could contribute to the amelioration and reduction of symptoms and negative effects of MS. In this context, some authors addressed this topic and suggested at least three possible approaches, namely direct manipulation, indirect manipulation, and microbiota replacement [67]. Direct manipulation is generally focused on the supplementation of probiotics. Some studies on probiotic reported positive effects after the supplementation of *Limosilactobacillus reuteri*, or of a cocktail including several lactobacilli and bifidobacteria (e.g., *Lactobacillus acidophilus*, *Lactiplantibacillus plantarum*, *Lacticaseibacillus paracasei*, *Bifidobacterium breve*, *B. infantis* etc.) at least on animal models [73,74,75], and other positive results are reported by some clinical studies [67]. A second approach for direct manipulation is an antibiotic treatment to deplete pro-inflammatory taxa in the gut, but this method has shown conflicting results [67].

Indirect manipulation is focused on the modification of the gut microbiota of MS patients through dietary habits, by promoting butyrate-producing bacteria (for example, through the supplementation of prebiotics) [67]. Finally, microbiota transplantation seems a promising approach, at least based on some pilot studies [76,77].

It is worth mentioning that microbiota modulation has been recognized as a possible approach to halting neurodegenerative symptoms in MS patients, but also as a method to prevent disease appearance by assessing positive biomarkers or as a means to promote active remyelination, considering the positive outcome of butyrate-producing bacteria on animal models [78].

In this context, the discovery of new biomarkers is crucial. Nowadays, there are three main kinds of biomarkers in MS, known as diagnostic biomarkers (to distinguish patients with MS from healthy controls), disease activity biomarkers (to assess inflammation, oxidative stress, or demyelination as well as disease progression), and treatment response biomarkers (to study the effect of a therapy) [79]. A drawback is the lack of a predictive or early-diagnosis biomarker that can identify individuals at risk of developing MS or point out neurologically asymptomatic individuals. An omics approach could be useful through the integration of data and technology from several methodologies (genomics—mainly next-generation sequencing or genome-wide association studies—transcriptomics, lipidomic, and proteomic) [80].

Another promising way is the possibility of developing a new series of non-invasive biomarkers based on saliva or urine due to the promising results of some authors in elucidating the potential of some molecules (human leucocyte antigen-class II; immunoglobulin free light chains; urinary proteins) [81,82,83].

## 6. MS Patients, Abdominal Pains, and Probiotics: A Case Study

As reported in previous sections, MS patients generally suffer from a condition of dysbiosis in their gut microbiota, and this condition could be associated with mild or severe gastro-intestinal symptoms. Gut pains are becoming more important as experts and patients become more aware of the role of gut microbiota, but the true weight of this factor, according to the authors, is still underestimated.

Thus, a questionnaire was distributed to people with MS in order to obtain an accurate estimate of gastrointestinal symptoms caused by intestinal dysbiosis as well as assess their willingness to manipulate their microbiota with probiotics. Other additional data recovered were on dietary habits (protein and fiber consumption) and the form of MS.

The questionnaire was composed of 20 questions and was submitted through Google Forms to target people affected by MS in Foggia County. After form completion, data were collected, modeled as frequencies, and/or submitted to multiple regression procedures to highlight possible correlations.

**Figure 3:**
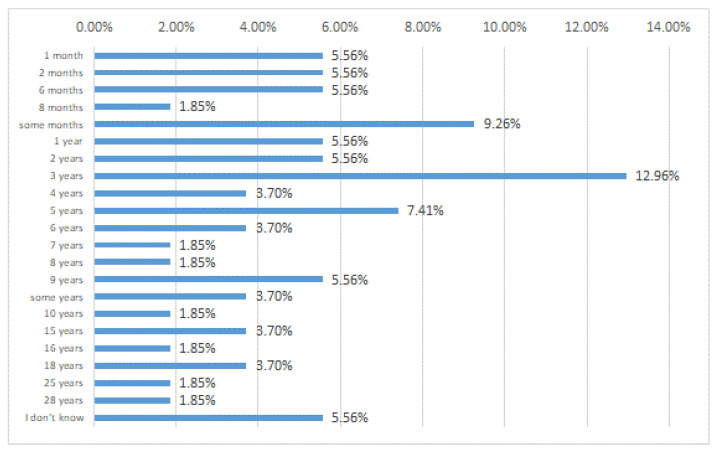
When did you receive your first diagnosis?

The questionnaire was completed anonymously by 55 subjects (12 males and 43 females). The first result was a higher prevalence of MS in people born in 1983. An interesting result emerged from the second question, which aimed to know how much time elapses between the onset of the disease and the medical diagnosis. The long time between the first symptoms and the medical diagnosis seems to be a real problem. In particular, 11% of volunteers had served for at least three years. This problem could be caused by incomplete diagnostic protocols, and this seems to be the first and most important problem in coping with the disease. Concerning the first diagnosis (Figure 3), 13% of interviewees reported 3 years, 7.41% reported a time up to 5 years, and 37.02% indicated more than 5 years.

**Figure 4:**
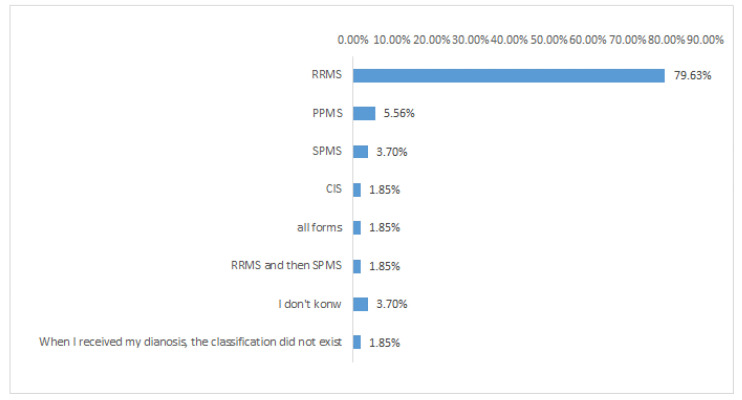
Types of multiple sclerosis among volunteers. RRMS, relapsing remitting multiple sclerosis; PPMS, primary progressive multiple sclerosis; SPMS, secondary progressive multiple sclerosis; CIS, clinically isolated syndrome.

The following question was aimed at highlighting the most prevalent type of MS, and the collected answers (Figure 4) revealed a significantly higher prevalence of RR-MS (78.2%) *versus* all the other MS types. This result is according to the scientific literature; in fact, the most common MS type is the relapsing-remitting one, regardless of age and gender. As reported by several scientific studies, an early diagnosis should be the first tool to define and control the disease.

**Figure 5:**
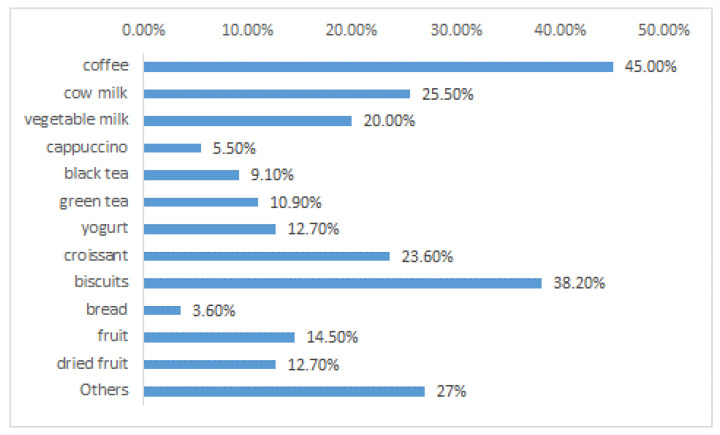
General eating habits of the tested people (What do you usually eat or drink for breakfast?) You can choose more options.

The questions numbered from four to six were aimed at collecting information on the general eating habits of the subjects (breakfast, lunch, any snacks, alcohol consumption, etc.). For example, Figure 5 shows what volunteers usually eat for breakfast; most interviewees (45%) drink coffee. Others eat biscuits (38.2%), croissants (23.6%). Healthier foods, functional for gut microbiota, are consumed by a small part of the sample; for example, only 12.7% of people usually eat yogurt.

The last seven questions were aimed at estimating the frequency, nature, and psycho-physical impact of gastrointestinal symptoms that may be related to dysbiosis.

**Figure 6:**
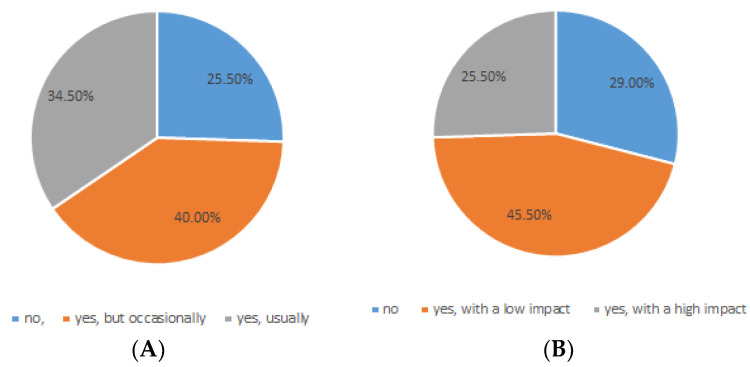
Percentage of subjects suffering from gastrointestinal symptoms. (**A**) Have you had gastro-intestinal symptoms? (**B**) Do gastro-intestinal symptoms affect your well-being?

An interesting result was that ca. 75% of subjects reported gastrointestinal disorders (Figure 6); constipation (47.3%), abdominal bloating (45.5%), and diarrhea (25.5%) seem to be the most prevalent symptoms, followed by abdominal swelling. Furthermore, most of the subjects hypothesized a correlation between gastrointestinal symptoms and the intake of certain foods. The statistics did not show a correlation between the forms of MS and gastro-intestinal symptoms (*p* > 0.05), thus suggesting that dysbiosis could be a problem for all forms; however, the data collected for some forms of MS require further investigations to corroborate this result (few responses to the questionnaire).

**Figure 7:**
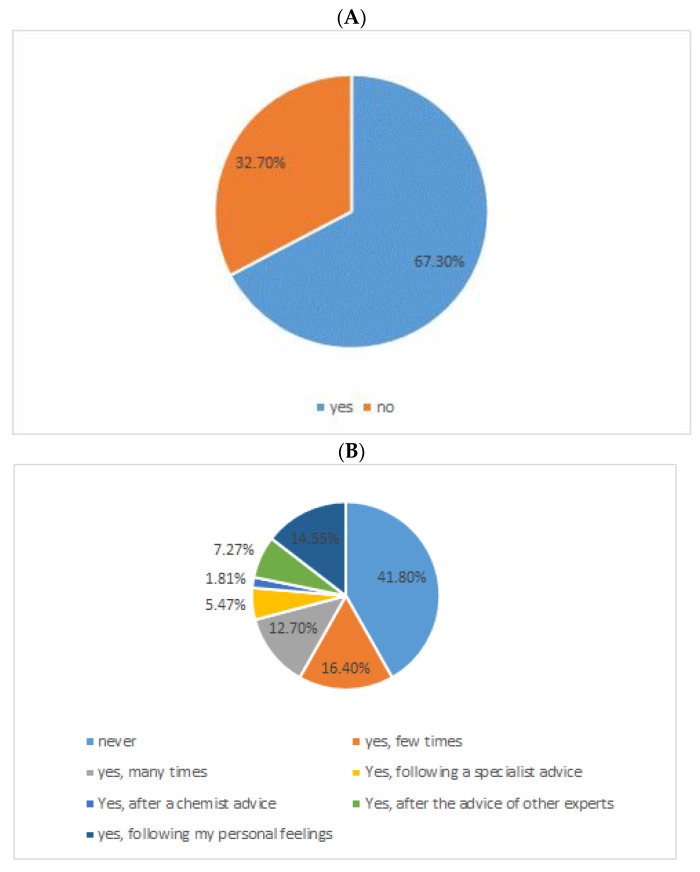
Awareness about probiotics and the use of probiotic foods. (**A**) Do you know what probiotics are? (**B**) Have you ever used probiotics?

**Figure 8:**
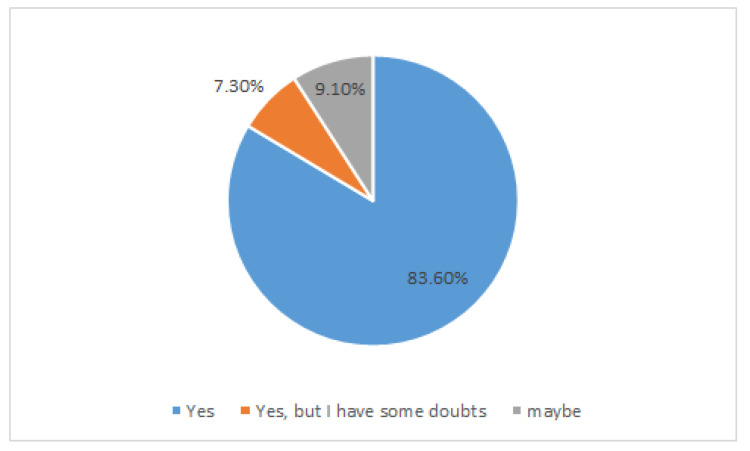
Willingness to use probiotics (Would you consume probiotics if research showed that they had an effective benefit for your disease?).

Regarding probiotics, from the collected data we find that more than 67.3% of all interviewees are informed about probiotics (Figure 7A) and 58.2% “use” them (Figure 7B). 14.5% took probiotics as directed by the neurologist or other health professionals; 27.2% chose to take probiotics on the basis of their general knowledge (Figure 7B). But the most significant data emerged from the last and conclusive question of the questionnaire, which aimed to highlight the willingness to use probiotics with proven beneficial effects on the clinical manifestations of the disease. It’s worth noting that 83% believe there’s a link between gastrointestinal symptoms and overall health (Figure 8).

## 7. Conclusions

Many metabolites derived from gut microbiota have immunomodulatory properties; these metabolites interact with immune system cells by activating pro- or anti-inflammatory responses. These metabolites derive from the essential amino acid Trp, and among these, kynurenine and indole substantially show pro-inflammatory effects, while serotonin, IPA, IAId, and IAA have an anti-inflammatory action; other compounds are synthesized from fibers (SCFA).

Physio-pathological relationships between gut microbiota and the immune system have already been established, and it is plausible to think that a particular condition of intestinal dysbiosis is linked to an auto-immune pathology such as MS.

Furthermore, the studies that compared the gut microbiota of healthy individuals and others with MS have found that subjects with MS showed a significant reduction in the variety and number of bacterial genera able to produce high amounts of SCFA (i.e., *Bacteroides*, *Parabacteroides*, and *Prevotella*).

A growing spread of MS among young adults and the autoimmune nature of this disease, affecting the central nervous system (CNS), have been recently studied, and scientific explanations of the two-way communication between the gut microbiota and the CNS have been demonstrated. However, further and more detailed studies are needed to be able to correlate some enterotypes (i.e., enterotype 3) with subjects affected by MS.

Finally, the questionnaire stressed the importance of gastro-intestinal symptoms in MS patients for well-being and quality of life; the data indicate a significant incidence of gastrointestinal disorders that impact the general health conditions of patients and their wellbeing.

A coordinated information and education action should be necessary to promote "food awareness," improve eating habits, and prevent dysbiosis as the cause of gastrointestinal disturbances.

The diagnosis is the most critical factor, requiring too much time. Nevertheless, once the diagnosis is made, effective food education and the use of functional probiotics may be considered some interesting tools useful to improve the condition of MS patients (the symptoms and course of the disease), also by means of gut microbiota modulation.

## Supplementary Materials

The following supporting information can be downloaded at: https://www.mdpi.com/article/10.3390/nu15040942/s1. Table S1: Composition of human gut microbiota.
