# Supplementary material for: Gut-Microbiota, and Multiple Sclerosis: Background, Evidence, and Perspectives
Source: Nutrients. 2023 Feb 14;15(4):942. doi: 10.3390/nu15040942 (PMC9965298; doi:10.3390/nu15040942)
Supplement: Supplementary file 1 [file nutrients-15-00942-s001.zip › nutrients-2185864-supplementary.pdf]

Table S1. Composition of human gut microbiota.

PHYLUM	Relative abundance	Genera and species	Main functions
Firmicutes	60-65%	*Clostridium*<br>*Eubacterium*<br>*Faecalibacterium*<br>*Lactobacillus*<br>*Roseburia*<br>*Ruminococcus*	Fiber fermentation and butyrate production
Bacteroidetes	20-25%	*Alistipes*<br>*Bacteroides*<br>*Parabacteroides*<br>*Prophyromonas*<br>*Provotella*	Catabolism of polysaccharides<br><br>Fiber fermentation and butyrate production
Proteobacteria	5-10%	*Escherichia coli*	
Actinobacteria	3%	*Bifidobacterium*<br>*Colinsella*	Synthesis of vitamins
Archea	<1%	*Methanobrevibacter*<br>*Methanosphaera*	Methane production
Deferribacteres	<1%		Iron degradation
Fusobacteria	<1%	*Fusobacterium nucleatum*	Tumorigenic factor with proinflammatory properties
Melainabacteria	<1%		Production of vitamins B and K, fermentation of carbohydrates, synthesis of ethanol, lactate and formate
Spirochaetes	<1%	*Treponema*	
Verrucomicrobia	<1%	*Akkermansia muciniphila*	
